# Supplementary material for: Transcriptome Analysis Reveals Vimentin-Induced Disruption of Cell–Cell Associations Augments Breast Cancer Cell Migration
Source: Cells. 2022 Dec 13;11(24):4035. doi: 10.3390/cells11244035 (PMC9776984; doi:10.3390/cells11244035)
Supplement: Supplementary file 1 [file cells-11-04035-s001.zip › DEGs and enrichment analysis.zip!/DEGs and enrichment analysis/WT_VIMvsVector KEGG/ALL/WT_VIMvsVector_ALL.pathway_map.zip!/WT_VIMvsVector_ALL.pathway_map/src/hsa03013.html]

hsa03013


- hsa:10189

- Up regulated genes

ENSG00000183684(1.51329373488818)

- hsa:79897

- Up regulated genes

novel.674(3.66392133841979)

- hsa:10460

- Up regulated genes

ENSG00000013810(1.81695285068003)

Close
